# The role of prediction and visual tracking strategies during manual interception: An exploration of individual differences

**DOI:** 10.1167/jov.24.6.4

**Published:** 2024-06-06

**Authors:** Tom Arthur, Samuel Vine, Mark Wilson, David Harris

**Affiliations:** 1 School of Public Health and Sport Sciences, Medical School, University of Exeter, Exeter, EX1 2LU, UK

**Keywords:** eye tracking, vision, skill acquisition, interception

## Abstract

The interception (or avoidance) of moving objects is a common component of various daily living tasks; however, it remains unclear whether precise alignment of foveal vision with a target is important for motor performance. Furthermore, there has also been little examination of individual differences in visual tracking strategy and the use of anticipatory gaze adjustments. We examined the importance of in-flight tracking and predictive visual behaviors using a virtual reality environment that required participants (*n* = 41) to intercept tennis balls projected from one of two possible locations. Here, we explored whether different tracking strategies spontaneously arose during the task, and which were most effective. Although indices of closer in-flight tracking (pursuit gain, tracking coherence, tracking lag, and saccades) were predictive of better interception performance, these relationships were rather weak. Anticipatory gaze shifts toward the correct release location of the ball provided no benefit for subsequent interception. Nonetheless, two interceptive strategies were evident: 1) early anticipation of the ball's onset location followed by attempts to closely track the ball in flight (i.e., predictive strategy); or 2) positioning gaze between possible onset locations and then using peripheral vision to locate the moving ball (i.e., a visual pivot strategy). Despite showing much poorer in-flight foveal tracking of the ball, participants adopting a visual pivot strategy performed slightly better in the task. Overall, these results indicate that precise alignment of the fovea with the target may not be critical for interception tasks, but that observers can adopt quite varied visual guidance approaches.

## Introduction

We interact with numerous moving objects daily, such as when we attempt to catch a ball, shake someone's hand, or avoid collisions in a busy shop. Even when target items are still or stable, we may be in motion relative to them (e.g., when picking up your keys as you leave the house). For these tasks, dynamic motor actions are guided by salient visual cues and the oculomotor system's remarkable capacity to perceive moving targets (Fooken, Yeo, Pai, & Spering, 2016, Fooken, Yeo, Pai, & Spering 2021; Land, 2006; Sugar, Mcbeath, & Wang, 2006). However, it remains controversial whether precise tracking of a visual target is *required* to perform successful interceptions. On one hand, there is evidence that expertise is closely linked to better tracking abilities in sport (Bahill & LaRitz, 1970; Mallek, Benguigui, Dicks, & Thouvarecq, 2017) and that shortened tracking durations can impair motor control and planning (Sun, Zhang, Vine, & Wilson, 2016; see also de la Malla & López-Moliner, 2015). Yet, other studies have shown only small differences in the ability of expert and novice players to pick up visual information (Abernethy, 1990; Harris, Wilson, Crowe, & Vine, 2020; Rodrigues, Vickers, & Williams, 2002) and that tracking can vary considerably in duration and quality across subjects of the same skill level (Croft, Button & Dicks, 2010; Oudejans, Michaels, Bakker, & Davids, 1999; Ramsey et al., 2020; Singer et al., 1998). Moreover, in some interceptive tasks, the target is moving too fast to be tracked accurately using smooth pursuit eye movements, and so predictive saccades must be used (Orban de Xivry & Lefèvre, 2007). These anticipatory gaze behaviors generally shift the point of gaze ahead of a moving target, toward its future projected position. For example, when intercepting a bouncing ball in cricket, saccades are made to its expected future location, which highly skilled players do earlier and more accurately (Land & McLeod, 2000; Mann, Spratford, & Abernethy, 2013). Hence, successful interception may not necessarily rest on precise visual tracking abilities.

There is wide theoretical dispute around the role of predictive models versus the sufficiency of continuous coupling to visual information in interceptive tasks (Gray, 2009; Katsumata & Russell, 2012; Peper, Bootsma, Mestre, & Bakker, 1994; Zago, McIntyre, Senot, & Lacquaniti, 2009). Approaches focusing on internal generative models posit that the brain uses constantly updated expectations to extrapolate the most likely future motion of the target to supplement the available visual information (McIntyre, Zago, Berthoz, & Lacquaniti, 2001; Parr, Sajid, Da Costa, Mirza, & Friston, 2021; Zago et al., 2004). Indeed, research has shown that, when the trajectory and bounciness of balls changes during interception tasks, individuals will adapt their eye movements to anticipate the new most likely flight paths (Arthur et al., 2021; Arthur & Harris, 2021; Diaz, Cooper, & Hayhoe, 2013). By contrast, explanations focusing on prospective control suggest that the brain uses only continuous sensory information to regulate action, and not internal predictive models (Gibson, 1979; Lee, 1998). Although this continuous provision of visual information could still occur via anticipation-like gaze patterns, this view emphasizes a fundamental importance of online tracking behaviors and visuomotor mapping during actions (see Gray, 2021). Therefore, although performers must clearly anticipate the behavior of the visual targets during time-constrained interceptive tasks (either through model-based predictions or prospective online control), important questions remain about how successful anticipation might influence tracking behaviors. For instance, does a successful prediction about the early motion of a projectile facilitate closer subsequent visual tracking? Or does it simply serve to reduce the demands and/or need for further tracking operations?

Notably, most research in the field has sought to understand the complex underlying mechanisms of visuomotor control via simple novice versus expert study comparisons (Abernethy, 1990; Mallek et al., 2017; Rodrigues et al., 2002). Indeed, although sensorimotor behavior and predictions involve highly individualized and context-sensitive processes (which can be systematically controlled over time) (Arthur & Harris, 2021), relatively few investigations have addressed the dependence of outcome success on the quality of tracking within individuals. An exception here is a study by Cesqui, Mezzetti, Lacquaniti, & d'Avella, (2015), who examined whether keeping the eyes on the ball (i.e., foveating the target) is crucial for good performance in a one-handed catching task. In this study, a positive relationship was shown between pursuit duration and catching performance, but many other aspects of visual tracking were not related to success. As such, the authors concluded that factors unrelated to eye movements may underlie the observed differences in interceptive performance.

The present study aimed to extend the work of Cesqui et al. (2015) by exploring the underlying mechanisms that determine interceptive motor abilities. To do this, we developed a manual interception task in virtual reality (VR) (see Harris, Vine, Wilson, & Arthur, 2022), which could illustrate the spontaneous tracking behaviors that are adopted during representative, movement-based activities. The paradigm was designed to decipher between predictive gaze responses (that are context sensitive and indicative of an internal generative model) and the continuous, online mapping of visual information in a manner that is rarely possible in real-world environments. Specifically, participants were required to intercept a fast-moving ball, which would travel toward them from one of two possible locations. This task has parallels with the return of serve in squash or tennis, in that a performer can rely on predictions about a ball's likely direction of travel and in-flight trajectory cues (Hayhoe, McKinney, Chajka, & Pelz, 2012). However, by systematically manipulating the ball's release location over time, the virtual task environment also introduced a degree of probabilistic uncertainty that is not present in many traditional laboratory assessments (e.g., Cesqui et al., 2015). Such conditions could highlight whether interception performance was linked to a person's gaze tracking accuracy and/or dynamic state predictions (e.g., their expectations about likely ball release locations, the uncertainty of these estimates, and the surprise associated with statistically unlikely observations).

Crucially, and in contrast with most existing research in the field, experimental conditions were performed in an unconstrained task that would demonstrate spontaneous visuomotor responses. Given that highly varied visuomotor strategies can often emerge for these type of skills (Croft et al., 2010; Dicks, Davids, & Button, 2010, Dicks, Davids, & Button, 2017; Ramsey et al., 2020), we also sought to explore the different strategies that were adopted by participants, and whether these might account for some of the factors unrelated to eye movements’ identified in Cesqui et al. (2015). Taken together, this approach allowed us to focus on the following research questions:

1. What visual tracking behaviors are related to interceptive performance?
2. Does successful prediction of release location enhance interceptive performance?
3. Do performers adopt similar tracking strategies, or do different approaches spontaneously emerge?

## Methods

### Design

This paper reports a secondary data analysis of an existing experimental data set and should, therefore, be treated as exploratory and hypothesis generating, rather than confirmatory. In an accompanying paper, we examined how participants learned the most likely projection location of the ball, but did not examine how participants actually tracked this target cue (Harris et al., 2023). The present analysis thus takes a more applied approach, which attempts to examine the functional role of predictions and continuous gaze mapping at an individual sensorimotor level. Here, we report on the different tracking strategies that were used and their relationship with interceptive success. Supplementary analyses have also been performed to explore the role of probabilistic uncertainty (https://osf.io/prq4c), expectedness (https://osf.io/x6ksh), and handedness (https://osf.io/7x6fu) on these individual behaviors.

The scope of such enquiry was designed to bridge the gap between computational and applied neuropsychology, by adopting a highly controlled task and sensory environment while allowing spontaneous and unconstrained visuomotor responses to emerge (as have been associated with performance expertise and/or success in previous research studies: e.g., Land & McLeod, 2000; Mann et al., 2013). Although many of the virtual task requirements and skill components differed from those in most typical real-world operations, it was designed to promote general gaze patterns and spatiotemporal characteristics that are akin to natural sporting interceptive actions (such as anticipatory saccades and fixations, followed by the active monitoring of a moving visual target) (see Hayhoe et al., 2012; Land & McLeod, 2000). Hence, the study dataset was deemed uniquely suitable for addressing our a priori research questions in a manner that is meaningful for future investigations and applied practice.

### Participants

Forty-one participants (aged 18–44 years; mean, 24.2 ± 7.4 years; 17 males, 24 females) were recruited from the staff and student population at a UK university. Three participants self-reported as left handed (and held the VR controller in their left hand). Participants were naïve to the exact aims of the experiment. They attended a single session of data collection lasting approximately 45 minutes and were compensated £20 for taking part. Informed consent was obtained in accordance with British Psychological Society guidelines, and the study received approval from the Departmental Ethics Committee (University of Exeter, UK). The study methods closely adhered to the approved procedures and the Declaration of Helsinki. Because this was a secondary data analysis, the sample size could not be determined a priori.

### Task and materials

The experimental task consisted of a simplified racquetball game where participants were instructed to intercept a ball that was projected from one of two possible locations at the front of a virtual court. From one location to the other spanned 21.5° of visual angle, so there was no possibility of keeping both locations in foveal or parafoveal vision. Participants held a virtual racquet in their dominant hand, which was operated by the VR handheld controller. Balls were 5.7 cm in diameter and had the visual appearance of a real-world tennis ball. The virtual racquet had the dimensions 0.6  ×  0.3  ×  0.01 m, but the physical diameter of its collider surface was exaggerated by 20 cm to facilitate the detection of ball-to-racquet collisions.

**Figure 1.**
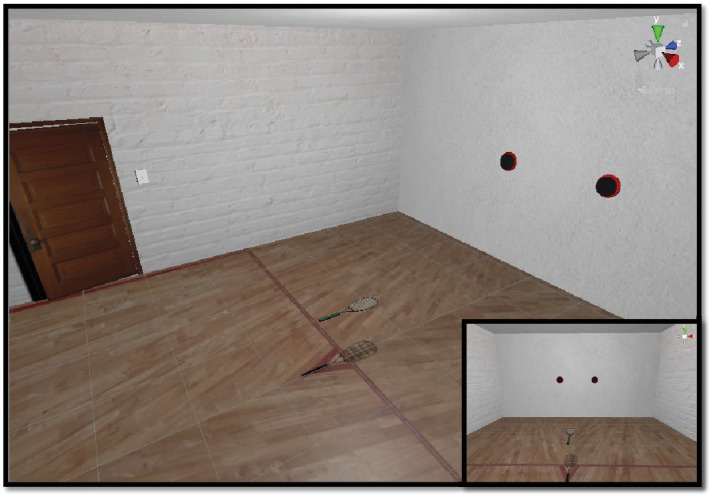
Virtual reality task environment. Participants stood on the red triangle marked in the center of the red line on the floor. The ball was projected from one of the two locations on the front wall. The ball passed the player without bouncing and they were instructed to intercept it with the racquet. The inset shows the player's view from inside the headset.

The virtual court was 8 × 8 m in size. Participants stood at the center of a red line, which indicated the midpoint between the front and back of the court ( Figure 1). This starting point was marked with a red triangle on the floor and the participants were instructed to return to this point at the start of each trial. This point was also marked on the laboratory floor so the experimenter could ensure that the participants returned to the correct location. Balls were projected at a speed that averaged 10 m/s over the 4 m between the release location and the red line, giving participants 0.4 seconds to react. Participants’ observation height corresponded with their own height (i.e., distance from the floor to the headset).

The virtual environment was developed using the gaming engine Unity (v2019.3.1f; Unity Technologies, San Francisco, CA) ( Figure 1) and was presented on an HTC Vive Pro Eye head-mounted display (HTC Inc., Taoyuan City, Taiwan), a consumer-grade VR system that has proven valid for small-area movement research tasks (field of view, 110 °; accuracy, 1.5 cm; jitter, 0.5 mm; latency, 22 ms) (Niehorster, Li, & Lappe, 2017). Movements of the headset and hand controller are sensed using inside out tracking and two infrared lighthouse base stations, which act as a reference point. The VR headset has inbuilt eye-tracking, based on binocular dark pupil at 120 Hz (spatial accuracy, 0.5°–1.1°; latency, 10 ms).

### Procedure

Participants attended the testing laboratory for a single visit that lasted approximately 45 minutes. After completing an informed consent form, they were fitted with the VR headset. The eye trackers were calibrated at the start of the experiment and again on any obvious displacement of the headset. Participants completed six practice trials to familiarize themselves with the task. On each trial, participants begun in the center of the court, standing on the short line ( Figure 1). The appearance of each ball was cued by three auditory tones, which were followed by a variable onset delay (a 0- to 5 -second window) that was randomly selected from a uniform distribution. The variable ball onset was used to increase the level of challenge (see report on pilot testing: Harris et al., 2022). The ball was projected to either the left or right side of the participant and reached them on the full (i.e., without bouncing) at around chest height (1.36 m). Participants were told to simply intercept the ball and that they did not have to hit it back in any particular direction. When the ball was intercepted with the racquet, it disappeared and a rewarding ding sound was played, alongside a haptic vibration from the handheld controller. If the ball was missed, an unrewarding buzz sound was played and no controller feedback was provided. Participants completed 120 test trials, split across 6 blocks of 20 trials each. The blocks varied in their distribution of left and right balls. The left/right splits of the blocks were 50/50 (2 blocks), 90/10, 70/30, 30/70, and 10/90. Participants completed these blocks in one of two pseudorandomized orders. For the main analyses (which examined the broad, functional role of predictions and continuous gaze mapping on interception performance), data were collapsed across each of the six trial blocks. However, block data were separated for our supplementary analyses of expectedness (https://osf.io/x6ksh), probabilistic uncertainty (https://osf.io/prq4c), and handedness (https://osf.io/7x6fu) variables.

### Measures

Eye-tracking data were recorded from the virtual environment and then processed using bespoke MATLAB scripts (MATLAB 2022b; MathWorks, Natick, MA), all of which are available at https://osf.io/tgx6r/. Both gaze-in-world coordinates (i.e., the intersection point of the gaze vector with the environment) and a single unit vector corresponding to cyclopean gaze direction were extracted. Data were denoised with a three frame moving median filter and then a second-order lowpass Butterworth filter (Fooken & Spering, 2020). Following recent recommendations (Cesqui, van de Langenberg, Lacquaniti, & d'Avella, 2013, Cesqui et al., 2015), different cut-off frequencies were applied for saccade identification (50 Hz) and analysis of positional tracking features (i.e., all other measures that were based on spatial gaze coordinate data; 15 Hz).

For calculation of the in-flight tracking metrics, cyclopean gaze direction and dynamic ball position data were plotted with respect to a two-dimensional head-centric vector in space (following Arthur et al., 2021; Diaz et al., 2013). Both the gaze and ball orientation vectors were taken from the origin of eye tracker and were aligned to the virtual racquetball environment (i.e., global Unity coordinates). Here, yaw angles represented rotation about a vertical axis that is in-line with gravity, with a value of 0° assigned to a vector that points straight ahead from the headset toward the front wall. Conversely, pitch values index angular deviance from a plane originating at eye-height that is parallel to the floor, such that a value of 90° will be assigned to vectors that points directly upwards and a value of −90° will be assigned to those that point straight down to the floor. From here, the following metrics were then calculated.

#### Tracking coherence and lag

To index the degree to which the movement of the eyes was coordinated with that of the ball, we calculated the cross-correlation between angular positions of the eye and ball in the yaw plane, which was the principal plane of movement of the ball relative to the observer. Cross-correlation is a measure of the similarity of two time series as a function of the displacement of one relative to the other. Specifically, it determines the extent to which the eye and ball yaw velocities covary, and the time lag at which this peak covariance occurs (see Laurutis, Niauronis, & Zemblys, 2010). This provides a measure of both tracking coherence (i.e., how closely did eye velocity in the yaw plane match that of the ball) and tracking lag (i.e., how far behind or ahead gaze was).

#### Saccades

During tracking, eye position can move in distinct jumps to either catch up with the target (Fooken et al., 2016, Fooken et al., 2021; Mrotek & Soechting, 2007) or to move ahead of it (Diaz et al., 2013; Land & McLeod, 2000; Mann, Nakamoto, Logt Sikkink, & Brenner, 2019). As in previous studies of interception (e.g., Arthur et al., 2021; Fooken et al., 2016; Mann et al., 2019), we calculated the number of saccadic movements that were executed during tracking periods of the task. Saccadic shifts were identified from portions of data where gaze acceleration exceeded five times its median absolute acceleration (Arthur et al., 2021; Mann et al., 2019). Gaze velocity had to exceed 40°/s for five consecutive frames and had to be at least 20% greater than that of the ball to avoid erroneous detections (e.g., due to pursuit or tracker noise artefacts). Time periods preceded by missing data were also excluded. Onset and offset times were determined from these signals using acceleration minima and maxima (Fooken & Spering, 2020).

#### Pursuit gain

Following Palidis, Wyder-Hodge, Fooken, & Spering, (2017), we calculated pursuit gain as the saccade-free eye velocity divided by target velocity. To do this, periods during which a saccade were made were first removed from the data. The gain value was then calculated for the closed-loop portions of tracking, which was defined as 140 ms after ball release, based on Fooken and Spering (2019). Closed-loop pursuit reflects tracking of the ball based on observed motion feedback (Palidis et al., 2017). Here, values close to one indicate that a visual target has been tracked very closely and continuously over time, whereas a value of less than one indicates that gaze has been rotating slower than ball, on average (in terms of relative angular positions).

#### Pre-onset prediction

**Figure 2.**
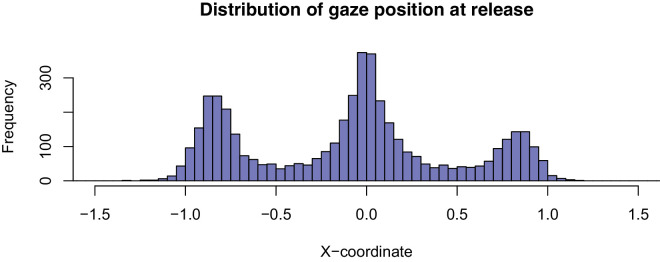
Distribution of predictive gaze position across all participants.

Finally, we calculated a variable to represent whether participants applied a correct sensorimotor prediction about projection location on each trial. Predictive gaze location was calculated as the horizontal gaze position at the termination of the auditory tones (averaged over a 50-ms window). Because the ball could appear at any time after the tone, this was taken as the critical moment for anticipating the projection location. Inspection of the data distribution plot (Figure 2) showed a clear multimodal distribution, with clusters forming around either of the two locations or in a central location. Gaze position was classified subsequently as either a clear prediction (on one of the two locations) or a nonpredictive (central) location. The central half of the space (10.9° of visual angle) between the two locations was treated as nonpredictive (from −0.4 to 0.4 in Figure 2, with the projection locations at −0.8 and 0.8).

### Data analysis

All variables were screened for outlying values more than 3 standard deviations from the mean, which were replaced with a Winsorized score. A series of linear mixed effects models (fitted using restricted maximum likelihood in the lme4 package) (Bates, Mächler, Bolker, & Walker, 2014) were then used to examine the relationship between tracking variables and interceptive performance. Model fit checks were performed using the ‘performance’ package (Lüdecke, Ben-Shachar, Patil, Waggoner, & Makowski, 2021). We report total *R*^2^ (variance explained by both fixed and random effects), marginal *R*^2^ (variance explained by fixed effects only), and standardized beta effect sizes for the mixed effects models. When evaluating the results, we follow Acock's (2014) rule of thumb for standardized beta effect size interpretation (i.e., that <0 .2 is weak, 0.2–0.5 is moderate, and >0 .5 is strong).

## Results

**Figure 3.**
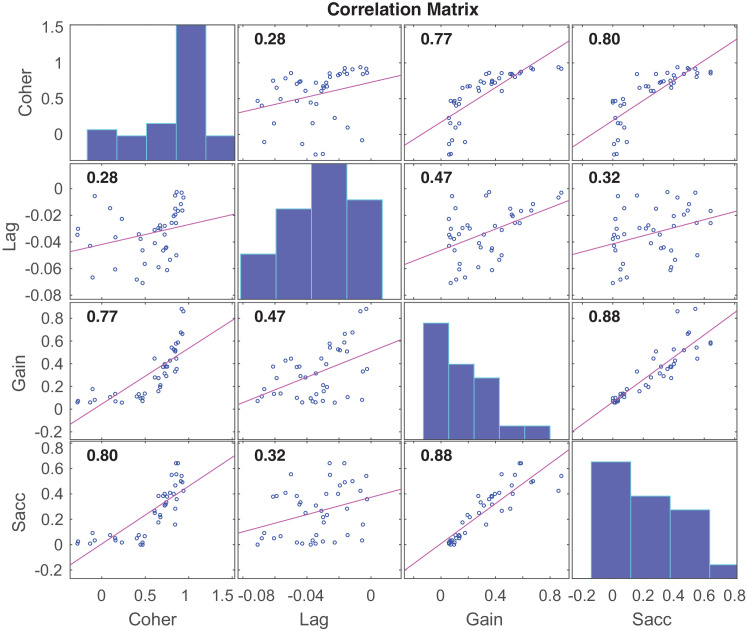
Correlation matrix showing relationships between the in-flight tracking variables. Panels on the diagonal illustrate the marginal distribution of each variable. Pink lines illustrate a fitted linear regression line. Correlation coefficients are shown in the top left of each panel. Coher, tracking coherence; Gain, tracking gain; Lag, peak tracking lag; Sacc, saccades.

First, we examined which of the tracking variables predicted interceptive performance in this task. Here, gaze data were collapsed across each of the six trial blocks, with supplementary analyses showing that none of the eye tracking metrics were significantly affected by the experimental changes in probabilistic uncertainty (see https://osf.io/prq4c). A correlation matrix of the relationships between variables is shown in Figure 3. Unsurprisingly, positive associations emerged between pursuit gain and the measures of tracking coherence, *R* = 0.77, *p* < 0.001, and lag, *R* = 0.47, *p* < 0.01). Notably, the average number of saccades was positively related to all three of these tracking measures (coherence: *R* = 0.80, *p* < 0.001; lag: *R* = 0.32, *p* = 0.04; and pursuit gain: *R* = 0.88; *p* < 0.001) (see Figure 3), indicating that a higher frequency of gaze shifts corresponded with a closer coupling between the fovea and ball.

### Tracking coherence

A mixed effects logistic regression model predicting interception success with tracking coherence as a fixed effect and participant as random effect had a total *R*^2^ of 0.30 and a marginal R^2^ of 0.002. Within this model the effect of tracking coherence was statistically significant but very weak, beta = −0.15, 95% confidence interval (CI) −0.29 to −8.28e-03, *p* = 0.04, standard beta = −0.09. The negative relationship indicated that higher coherence may be linked to poorer interception outcomes, although this weak relationship is clearly uncertain and requires further examination.

### Tracking lag

The mixed effects logistic regression model predicting interception success with tracking lag as a fixed effect and participant as random effect had a total *R*^2^ of 0.30 and a marginal *R*^2^ of 0.001. Within this model, the effect of tracking lag was statistically significant but again very weak, beta = 1.20, 95% CI 0.19 –2.21, *p* = 0.02; standard beta = 0.8). The positive relationship indicated that longer lags behind the ball led to poorer interception.

### Saccades

The mixed effects logistic regression model predicting interception success with saccades as a fixed effect and participant as a random effect had a total *R*^2^ of 0.29 and a marginal *R*^2^ of 0.003. Within this model, the effect of saccades was statistically significant but small, beta = −0.27, 95% CI −0.42 to −0.12, *p* < 0.001, standard beta = −0.13. The negative relationship indicated that fewer saccades related to better interception.

### Pursuit gain

The mixed effects logistic regression model with closed-loop gain as a fixed effect and participant as random effect had a total *R*^2^ of 0.30 and a marginal *R*^2^ of 0.001. Within this model, the effect of closed loop gain was narrowly significant but small, beta = 0.22, 95% CI 0.02 –0.42, *p* = 0.03, standard beta = 0.09, indicating higher gain values (i.e., closer to 1) were related to better interception.

### Combined model

**Figure 4.**
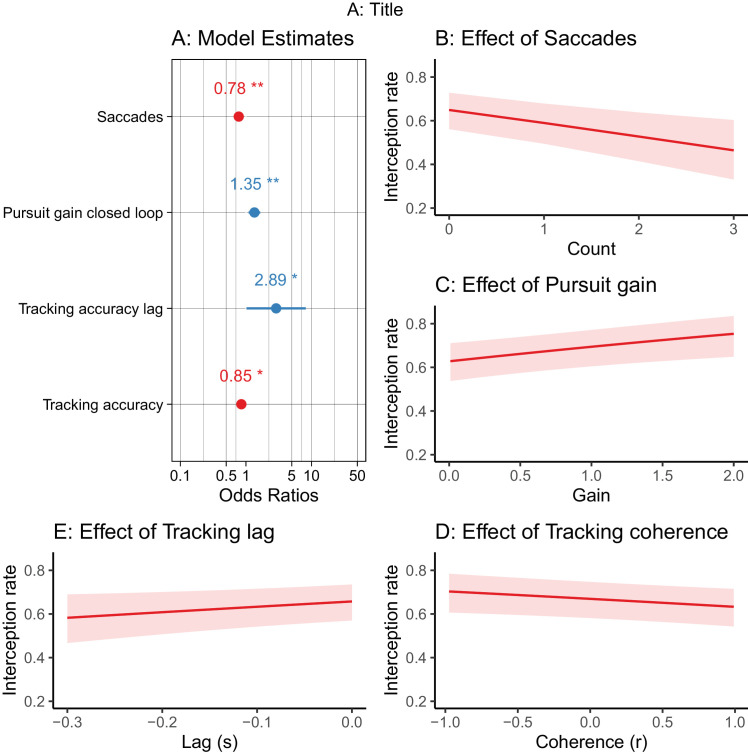
Results of full model. (A) Forest plot of all model predictors (significant predictors marked with a star, negative relationships are red and positive ones are blue). (B–E) The model estimated relationships for the effect of key predictors on interception.

Next, we entered all the tracking variables into a model simultaneously to examine which predictors remained significant in the presence of the other variables (Figure 4). The fitted model (with participant as random effect) had a conditional *R*^2^ of 0.30 and a marginal *R*^2^ of 0.01. Within this model, higher pursuit gain, beta = 0.30, 95% CI 0.09 –0.50, *p* = 0.004, standard beta = 0.12, and fewer saccades, beta = −0.25, 95% CI −0.41 to −0.10, *p* = 0.001, standard beta = −0.12, were the best predictors of success, but even these relationships were weak. Tracking coherence, beta = −0.16, 95% CI −0.30 to −0.02, *p* = 0.03, standard beta = −0.09, and tracking lag also remained statistically significant, beta = 1.05, 95% CI 0.01 –2.09, *p* = 0.047, standard beta = 0.07, but relationships were very weak.

### Prediction accuracy

**Figure 5.**
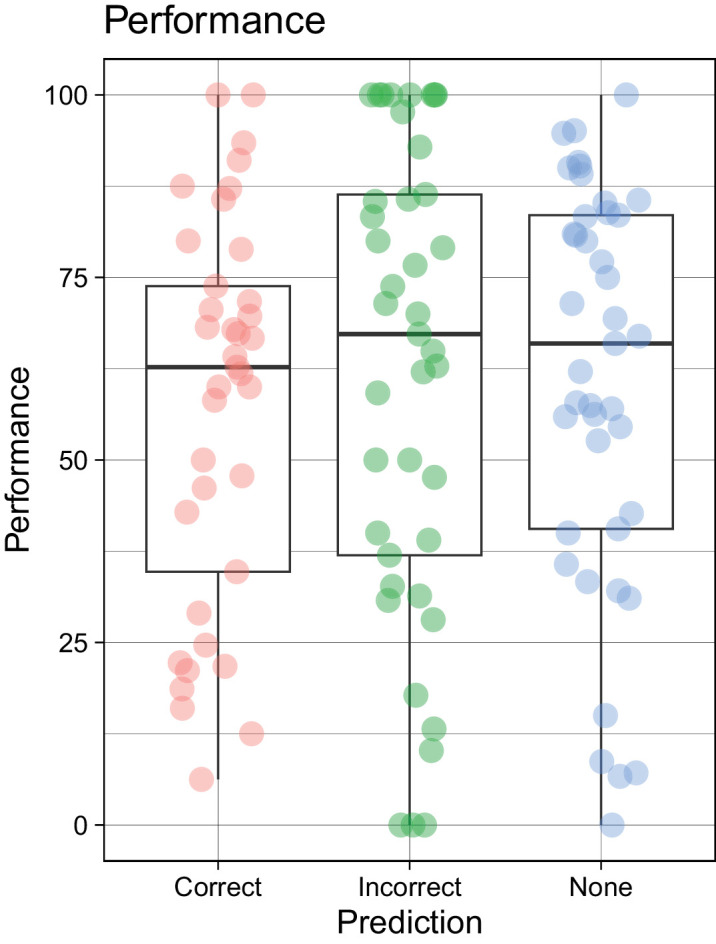
Boxplot with overlaid data points (individual participants) showing the comparison of interceptive performance for correct, incorrect, and no prediction trials.

To examine the effects of correctly predicting the ball release location, we fitted a mixed effects regression model to the interception data using prediction (correct or incorrect) as a predictor, with random slopes and intercepts for participant. Correct prediction was based on the location of gaze at the stimulus onset (end of beeps). Trials with nonpredictive gaze locations were not included in the model but are shown in Figure 5 for comparison. The model's total *R*^2^ was 0.34 and marginal *R*^2^ was 0.004. Within this model, the effect of prediction accuracy was statistically nonsignificant, beta = 0.28, 95% CI −0.04 to 0.59, *p* = 0.09, standard beta = 0.28, suggesting that it had minimal effects on interceptive motor performance.

### Individualized gaze strategies

Visual tracking behaviors may not just be indexed by singular metrics, like pursuit gain or the number of saccades, but rather how they cluster together to form global strategies. There is an increasing recognition that, even among groups of individuals who are highly experienced at a task, a range of strategies will emerge (Dicks et al., 2010; Ramsey et al., 2020). We, therefore, sought to understand the individual differences in visual tracking that existed in this study. Based on literature from sporting tasks, which has reported the use of both foveal and peripheral monitoring strategies in time-constrained tasks (Ramachandran, Watts, Jackson, Hayes, & Causer, 2021; Vater, Kredel, & Hossner, 2017, Vater, Williams, & Hossner, 2020), we anticipated that participants likely adopted one of two approaches before stimulus onset: 1) a predicting strategy, where the onset location of the ball on each trial is anticipated and gaze is directed toward the expected side, or 2) a visual pivot strategy, where the eyes are directed to a central location and foveal vision is used to locate the ball and then saccade toward it.

**Figure 6.**
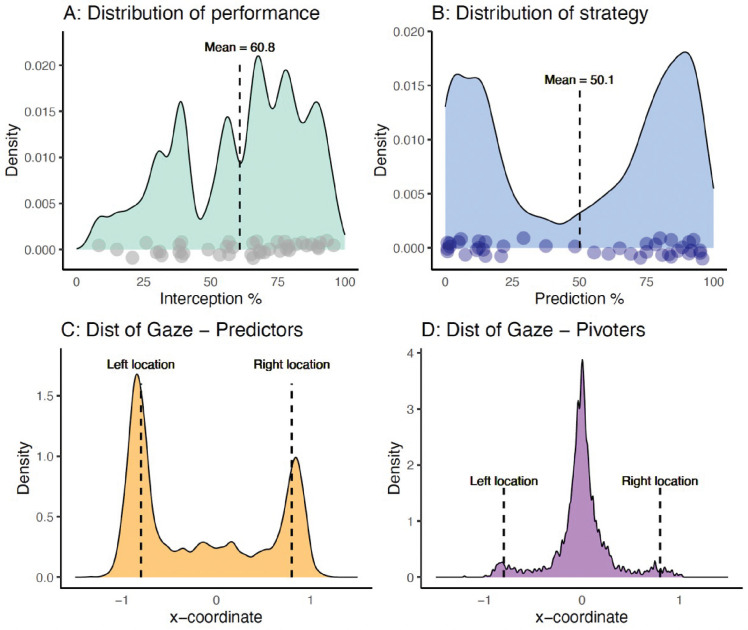
(A) Distribution of interceptive performance. (B) Distribution of predicting versus visual pivot strategies before onset. (C) Distribution of pre-release gaze position in the predictors group. (D) Distribution of pre-release gaze position in the visual pivoters group.

Based on our pre-onset eye tracking data, it was evident that gaze was predominantly directed to central and/or extreme spatial locations. Specifically, participants looked just outside (i.e., slightly ahead) of the virtual wall holes, or to a straight-ahead location that was in the middle of these ball release locations ( Figures 2, 6A, and 6B). As it was initially unclear whether participants used a single or mixture of approaches, we next calculated the percentage of trials in which each participant made a prediction versus located gaze centrally. The distribution of strategy data ( Figure 6B) indicated that performers adopted either a predictive or a visual pivot strategy, with few using a combination of the two.

**Figure 7.**
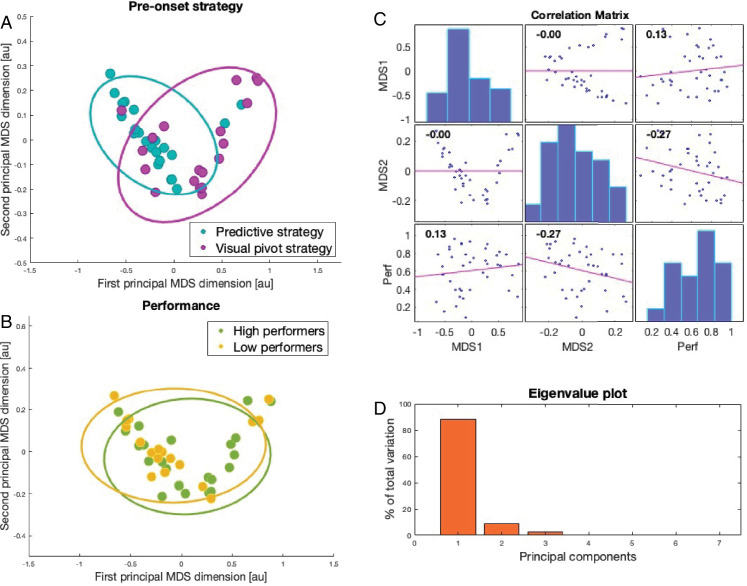
Results of the multidimensional scaling (MDS) analyses. (A and B) The first two MDS dimensions, where data points represent individual participants plotted in geometric space, so that dots closer to each other had more similar in-flight tracking strategies. Data points are color coded by pre-onset strategy (A) and performance (B), with plotted ellipses indicating 95% confidence intervals of the two-dimensional distribution. (C) Correlation plot of the relationships between the two principal MDS dimensions and interception performance, with *r* values marked in each subpanel. (D) Eigenvalues of the principal MDS dimensions. The first two dimensions explained 88% and 9% of the variance (sum 97%).

To explore how these initial, pre-onset gaze strategies might influence subsequent in-flight tracking behaviors, we adopted a multidimensional scaling (MDS) approach. MDS is a dimension reduction analysis technique that has been widely applied for visualizing the similarities and differences between data sets (Borg & Groenen, 2005). Crucially, it allows us to visualize the clustering of gaze strategies (i.e., combinations of pursuit gain, saccades, tracking), rather than examining singular metrics. MDS represents the tracking strategy of each participant as a point in an abstract geometric space, such that individuals who use similar tracking behaviors cluster together. It, therefore, allows us to identify principal dimensions that best explain the relationships between these strategies (see Słowiński et al., 2019 [Słowiński et al., 2022] for previous applications to visuomotor coordination and Harris et al. [2023] for application to characterizing visual search expertise). Figures 7A and 7B show the two principal MDS dimensions (i.e., the most significant dimensions of the abstract geometric space) with data points color-coded based on pre-onset strategy (Figure 7A) and high versus low performance (Figure 7B). The groupings based on strategy and performance were determined by a mean-split for the purposes of visualization. The results suggest that there was a distinct clustering of in-flight tracking behaviors for the predictive (*n* = 22) and visual pivot (*n* = 19) groups, indicating that different pre-onset strategies led to fundamentally different in-flight tracking profiles. Figure 7B, however, suggests that there was no clustering of tracking strategy for high versus low performers (almost total overlap), which implies that no particular tracking approach was associated with better performance.

**Figure 8.**
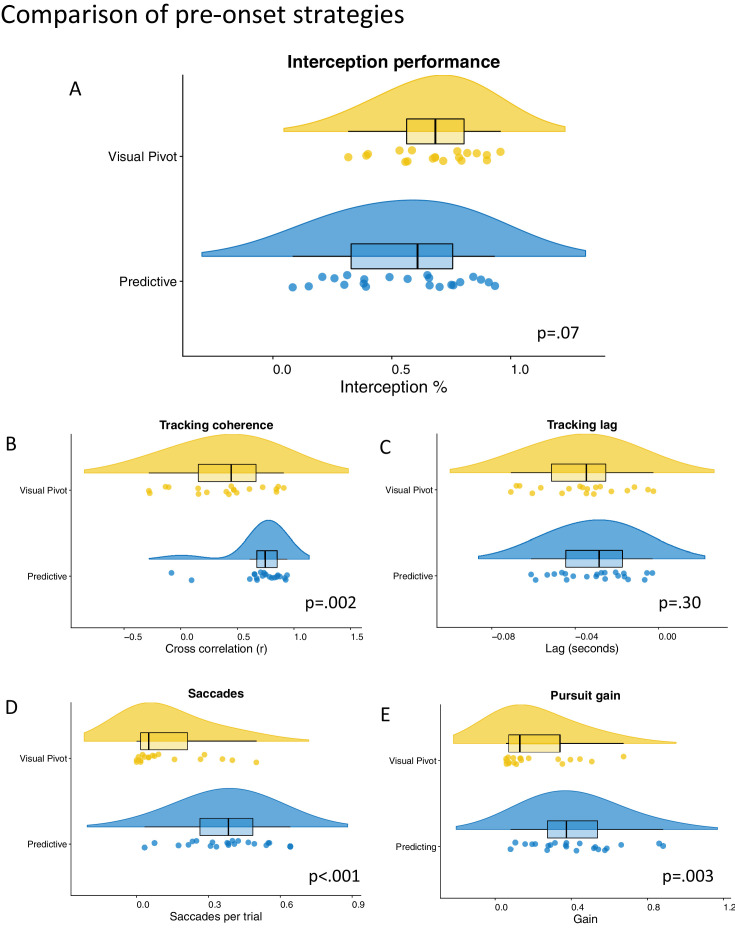
Raincloud plots of comparisons of tracking variables between those adopting predictive and visual pivot strategies.

To further examine the differences between the two global strategies, we performed a series of pairwise comparisons between the predictive and visual pivot groupings (see Table 1). These comparisons indicated that those adopting the visual pivot strategy showed better interception performance. This result did not reach statistical significance, *p* = 0.07, *d* = 0.58, but, given the large effect size, it is likely that this would have passed the threshold in a larger sample. The in-flight tracking of the ball by the visual pivot participants was much poorer, as shown by lower tracking coherence (Figure 8B) and pursuit gain values (Figure 8E). Visual pivot participants also exhibited fewer in-flight saccades (Figure 8D), with an average number of 0.13 ± 0.16 recorded per trial. These results suggest that those adopting a center-looking, visual pivot approach (before trial onset) did little to shift their gaze toward the ball after it was released (either through saccadic eye movements or smooth pursuit). Instead, they continued to rely on peripheral vision and made little attempt to track the ball in flight or adjust to its dynamic spatial position. This response was associated with accurate interception actions, although performance levels were poorer in low-probability trials (i.e., trials where the ball originated from a side that was less statistically likely; see the Supplementary Information). By contrast, those who applied pre-onset predictions about release location were able to execute more tightly coordinated in-flight tracking. Interestingly, though, this strategy did not transfer to better interceptive actions and may have even led to poorer task performance (especially for low-probability trials; see the Supplementary Information). In fact, this predictive group did not seem to benefit from using pre-onset gaze predictions at all, with post hoc *t* tests showing null differences in interception rate between their correct and incorrect prediction trials, *t*(21) = 0.08, *p* = 0.94, *d* = 0.01.

**Table 1.** Independent *t* test comparisons of performance and tracking variables between the two pre-onset strategies (predicting versus visual pivot).

	Statistic	*p* value	Cohen's d
Interception performance^*^	1.87	0.07	0.58
Tracking coherence^*^	−3.18	**0** **.002**	−1.03
Tracking lag	−1.04	0.304	−0.33
Gain	−3.17	**0** **.003**	−0.99
Saccades	−4.88	**<0.001**	−1.53

* Based on Welch's *t* test due to unequal variances.

Boldface entries indicate statistical significance.

## Discussion

This study sought to contribute to a better understanding of oculomotor control during interceptive motor actions. There is mixed evidence regarding the importance of precise foveal tracking during interception (Abernethy, 1990; Bahill & LaRitz, 1970; Harris et al., 2020; Mallek et al., 2017; Rodrigues et al., 2002; Sun et al., 2016), and potential individual differences in visual tracking behaviors (Croft et al., 2010; Oudejans, Michaels et al., 1999; Ramsey et al., 2020; Singer et al., 1998). This study significantly contributes to the field by addressing whether in-flight visual tracking features were an important predictor of performance in an interceptive task, and whether different visuomotor strategies spontaneously emerged. In short, results suggested that closer in-flight tracking of the target was not a strong predictor of interception in this task, and neither was correctly anticipating the projection location. Two clear visual guidance strategies emerged, which were characterized by either strong pre-onset prediction of the projection location followed by close in-flight tracking (predictive strategy), or a center-looking strategy that relied on peripheral vision (visual pivot strategy). Although these strategies influenced how individuals monitored the target visual cue, they had limited effects on overall interceptive performance.

Notably, none of the visual tracking variables in this study explained large amounts of variance in interceptive motor performance. Indeed, even for the overall model, which included all in-flight variables as predictors, the fixed effects explained only 2% of the total variance in performance. Higher pursuit gain values and fewer visual saccades were linked to better interception outcomes, which suggests that there may be some benefits from continuously tracking the ball during the selected movement task. However, these effects were small and performance was not impaired in participants who relied predominantly on peripheral vision (e.g., those using a visual pivot strategy). As such, the findings question the value of in-flight foveal tracking for some movement skills, and add to the mixed results regarding the need for continuous vision of dynamic target cues (Cesqui et al., 2015; Croft et al., 2010; Katsumata & Russell, 2012; Mallek et al., 2017; Orban de Xivry, Coppe, Blohm, & Lefèvre, 2013).

As expected, we found that visuomotor responses were influenced by a person's dynamic expectations about the trajectory of upcoming balls, although these expectations did not always impact task performance. Indeed, about half the participants clearly tried to anticipate the side that the ball would travel from in each trial and would then use these predictions to direct their gaze behaviors. Despite this, interception rates were not higher on correctly anticipated trials and there were numerous participants who did not display pre-onset anticipatory gaze profiles. Given the substantive previous work that has shown predictive mechanisms to be important for interception skills (Diaz et al., 2013; Hayhoe, Mennie, Sullivan, & Gorgos, 2005; Hayhoe et al., 2012; McIntyre et al., 2001; Zago et al., 2004), we speculate that task success was being determined largely by other task-specific visuomotor control factors. For instance, even when anticipating the projection location correctly, the projection speed of the ball may have meant that navigating the racquet to the right location at the right time still posed a considerable challenge. Notably, supplementary analyses showed that performances were impaired across the study sample when balls were projected from less-probable projection locations (i.e., from a side that was uncommon or unexpected within a block of trials). These results support previous sport (e.g., Loffing, Stern, & Hagemann, 2015; Navia, van der Kamp, & Ruiz, 2013) and psychophysics (e.g., Gottsdanker & Kent, 1978) experiments, which show that motor responses are superior in environments that are congruent with prior expectations and/or experience. Therefore, although the relationship between overt gaze predictions and performance may have been weak, performance was better for more predictable trials (for further discussion of these findings, see the Supplementary Material).

Participant's gaze responses in this dataset could be strongly grouped into two distinct strategies, which we termed predictive and visual pivot. These individual differences contrast with Cesqui et al. (2015), who reported a consistent sequence and timing of eye movement events across participants in a one-handed catching task. The distribution of gaze strategy indicated that few participants used a mixture of these approaches; they either opted to make a guess at the location of the upcoming ball, or to look centrally before each trial. The participants then displayed distinct groupings of visual tracking behaviors, once the ball had been released. Specifically, after initially directing gaze toward its anticipated projection location (or just in front of this point), the predictive strategy group attempted to track the ball using smooth pursuit and/or small saccadic eye movements. Conversely, those selecting a visual pivot approach track the flight of the ball poorly and make little attempt to shift their gaze from a generic central location, thus seeming to rely on peripheral vision instead.

The term visual pivot was chosen based on previous work that has identified the importance of peripheral vision in dynamic sporting tasks (Vater et al., 2017, Vater et al., 2020). For instance, in combat sports, fast attacks from the hands and/or feet are detected with peripheral vision, with foveal gaze often directed to the trunk (Milazzo, Farrow, & Fournier, 2016; Milazzo, Farrow, Ruffault, 2016; Piras et al., 2014). Similar use of peripheral vision has been reported when juggling balls (Huys & Beek, 2002), controlling a boat (Manzanares, Menayo, & Segado, 2017), detecting a drowning person (Page, Bates, Long, Dawes, & Tipton, 2011), or in games like tennis or squash (Afonso, Garganta, Mcrobert, Williams, & Mesquita, 2012; Vansteenkiste, Vaeyens, Zeuwts, Philippaerts, & Lenoir, 2015). Interestingly, neither gaze strategy proved detrimental to performance in this study, with the visual pivot group performing slightly better overall, *p* = 0.07, *d* = 0.58. However, given that the processing of high-speed target information via peripheral vision can be misleading and can introduce movement biases (Shapiro, Lu, Huang, Knight, & Ennis, 2010; Tynan & Sekuler, 1982), future research could explore the efficacy of these strategies within increasingly challenging or complex motor tasks.

The results reported here should be interpreted in light of the specific characteristics of the study task and generalized with caution. Indeed, a VR-based interceptive motor skill was selected because it was both a novel task, and also one that would impose familiar and unconstrained demands. Consequently, it elicited spontaneous behaviors built on previous experience and spatiotemporal patterns that are broadly representative of natural activities (e.g., evidence of anticipatory gaze adjustments and continuous tracking of ball position) (Hayhoe et al., 2012; Land & McLeod, 2000). However, no participants could be considered expert in this specific skill, which limits our ability to identify any optimal tracking strategies or behaviors. The exact tracking behaviors that were observed in this study are, therefore, clearly specific to this particular task and these behaviors cannot be easily generalized to real-world skills. There are, however, several implications of our findings that may have wider relevance for our mechanistic understanding of visuomotor control that are likely to transcend multiple skill domains.

First, results show that interception can be achieved very successfully with limited tracking of the ball flight, as previously illustrated by Cesqui et al. (2015). This finding may be related to the predictability of the visuomotor environment: the only source of uncertainty in our task was the cue projection location, whereas the in-flight trajectories of the ball remained consistent. As a result, it is possible that participants learned that the ball would arrive in one of two possible locations and that the importance of in-flight tracking was decreased. If in-flight deviations were introduced to this task in the future, it is possible that closer foveal monitoring would be required. Indeed, Fink, Foo, & Warren, (2009) demonstrated that experienced baseball players couple their catching movements to continuous online visual information when midflight ball trajectories are perturbed in VR. Similarly, gaze tracking profiles may be altered in the presence of wider contextual cues, such as knowledge about an opponent or biological motion information (which can guide anticipatory responses) (see Murphy et al., 2016). Hence, it is important to acknowledge the need for further research in this area, and the likely discrepancies that exist between different interceptive motor skills. Nonetheless, our results indicate that for highly predictable trajectories, limited tracking may be required.

Next, results showed that interceptive actions can be performed successfully in very different ways. There is a growing acknowledgement that a focus on optimal gaze control strategies may have diverted attention away from understanding important inter-individual variability (Dicks, Button, Davids, Chow, & van der Kamp, 2017). Our findings support this idea and illustrate that very different approaches can emerge and can be effective within even a simple interception task. Such variability should be considered in future theoretical work; as it seems to be inherently related to the use of predictions and/or continuous gaze tracking during motor actions. In addition, there has also been a focus on foveal gaze in much visuomotor control research due to the ease of measurement with eye tracking technology. However, our results suggest that more work is needed to understand how peripheral vision strategies (e.g., visual pivots and gaze anchors) contribute to dynamic movement skills (e.g., Klostermann, Vater, Kredel, & Hossner, 2020).

Finally, our results shed light on the importance of prior expectations in interception, which is consistent with a predictive processing perspective (e.g., Clark, 2013; Friston, 2010). Anticipatory gaze patterns were used by most participants in the study and appeared to support closer visual tracking ( Figure 8). However, overall performance levels were not enhanced in these individuals, nor were they elevated by ‘correct’ prediction responses. That said, we did observe expectation-related biases: indeed, even the participants that used minimal anticipatory gaze adjustments (e.g., those employing a visual pivot strategy) were less successful on unexpected trials (see the Supplementary Information). Active inference theories (e.g., Parr et al., 2021) can provide a compelling rationale for these observed behaviors, because both the predictive gaze strategy (which could reflect probabilistic generative models) and the visual pivot strategy (which acts to decrease uncertainty) represent effective methods of minimizing prediction error. In fact, the minimization of prediction error could explain how peripheral visual cues are processed by the brain (Daucé, Albiges, & Perrinet, 2020; Seth, 2014) and how motor performances are maintained despite uncertain sensory information and delays across the neuromuscular system (see Mann, 2019). Future research could aim to examine these explanations more closely, by monitoring how individual gaze responses align with models of active inference and/or prospective motor control iteratively over time (e.g., using moment-by-moment or trial-by-trial computations).

## Conclusions

The ability to intercept, or avoid, moving objects in our environment has attracted wide research interest because it is such a fundamental visuomotor capability. Yet many questions remain about the nature of visual guidance, the importance of foveal tracking, and the role of predictive and prospective control (Gray, 2009; Katsumata & Russell, 2012; Peper et al., 1994; Zago et al., 2009). The present results support the view that precise alignment of the fovea with the target may not always be important for interception skills, and that gaze anchors and pivots may be a valid tracking strategy for some visually guided actions. Several other factors are likely to be significant contributors to interception proficiency. Notably, the impact of trial expectedness on our data highlights the relevance of prior expectations and internal predictive models. Additionally, biomechanical aspects influencing movement speed as well as motivational factors may play crucial roles in determining success. Because pre-onset eye movement behaviors showed limited correlation with interception, it would be worthwhile to explore the involvement of covert attention in this context. In addition, despite our familiarity with interceptive tasks, human observers can adopt different visual guidance approaches when faced with a new challenge or situational context. Research should examine how these individualized gaze strategies interact with common predictive models and/or online control processes, to provide a shared mechanistic basis for diverse sensorimotor behaviors.

## Supplementary Material

Supplement 1
