# Supplementary material for: The role of prediction and visual tracking strategies during manual interception: An exploration of individual differences
Source: J Vis. 2024 Jun 6;24(6):4. doi: 10.1167/jov.24.6.4 (PMC11160954; doi:10.1167/jov.24.6.4)
Supplement: Supplement 1 [file jovi-24-6-4_s001.pdf]

Supplementary Information

The effects of 'Expectedness' on task performance and gaze tracking responses

To further examine individual visuomotor behaviours, we explored how task performance and gaze tracking varied across trials with expected and unexpected ball trajectories. Here, trial 'expectedness' was determined by the veridical probabilities of each study block. Specifically, trials were classified as unexpected if they were from a less-probable side (i.e., balls from the right in 90/10 or 70/30 split blocks and balls on the left in 10/90 or 30/70 split blocks) or expected if they were from the more-probable location (e.g., balls from the left in 90/10 or 70/30 split blocks, or balls on the right in 10/90 or 30/70 split blocks). Interception performance, tracking coherence, saccade frequency, and pursuit gain were compared between these two types of trial for both 'visual pivot' and 'predictive' strategy groups.

For task performance (interception rate), a 2 x 2 ANOVA indicated a general effect of expectedness [$F(1,39)=7.68$, $p=.009$, $\eta^2=.01$], but no significant group [$F(1,39)=2.77$, $p=.10$, $\eta^2=.06$] or interaction [$F(1,39)=0.00$, $p=.98$, $\eta^2=.00$] effects. This indicates that both groups performed better when the release location was more 'expected' and probabilistically likely. Such findings are particularly notable for the visual pivot group, who did not rely on anticipatory eye movements in the study, but still showed attention-related biases.

For tracking coherence, there was no significant effects of expectedness [$F(1,39)=1.30$, $p=.26$, $\eta^2=.001$], but there was a significant effect of group [$F(1,39)=10.42$, $p=.003$, $\eta^2=.20$]. There was no significant group-by-expectedness interactions [$F(1,39)=0.50$, $p=.48$, $\eta^2=.00$], with coherence scores consistently higher for predictive group participants (see Supplementary Figure 1). This suggests that the predictive group tracked the ball much more closely than the visual pivot group, regardless of whether balls were expected or unexpected.

For pursuit gain, there was a significant effect of expectedness [$F(1,39)=9.25$, $p=.004$, $\eta^2=.01$], a significant effect of group [$F(1,39)=11.25$, $p=.002$, $\eta^2=.21$], and a significant interaction effect [$F(1,39)=8.01$, $p=.007$, $\eta^2=.01$]. Follow-up t-tests showed that, for the predictive group, pursuit gain was significantly higher for the unexpected trials ($p=.003$, $d=.72$). During these trials, overall eye velocity was much closer to that of the ball, which presumably reflects a need to catch up with this dynamic visual cue. However, the visual pivot group showed null differences between expected and unexpected trials ($p=.77$, $d=.07$), indicating that task probabilities had no influence on pursuit gain for these individuals.

For number of saccades, there was a significant main effect of expectedness [$F(1,39)=7.19$, $p=.01$, $\eta^2=.02$], and a significant effect of group [$F(1,39)=16.83$, $p<.001$, $\eta^2=.26$], but no significant interactions [$F(1,39)=0.04$, $p=.85$, $\eta^2=.00$]. While a higher number of saccades were made in unexpected trials across our study sample, scores were generally much higher in predictive strategy group than the visual pivot group (see Supplementary Figure 1).

Finally, we explored whether participants in the predictive strategy group were attempting to track the ball closely (e.g., using smooth pursuit eye movements and small 'catch up' saccades) or whether they were using a saccade-based strategy (e.g., where large eye and head movements shift gaze ahead of the moving ball, towards a future projected location). To do this, we examined the total amplitude of saccades that were made by participants for each type

of trial. Results showed significant main effects for expectedness [$F(1,39)=7.72$, $p=.008$, $\eta^2=.02$] and group [$F(1,39)=15.01$, $p<.001$, $\eta^2=.24$], but no significant interactions [$F(1,39)=0.20$, $p=.66$, $\eta^2=.00$]. Specifically, while both groups made larger saccadic shifts on unexpected trials, the predictive group also made larger saccades overall (see Supplementary Figure 1). Initially, these findings may suggest that a saccade-based strategy was being employed by the predictive group. However, saccade amplitudes were still notably modest in the predictive participants, and these observed group differences are being largely driven by the extremely low visual pivot values. Firstly, such findings reinforce our conclusions that the visual pivot participants were rarely attempting to track the ball using foveal vision, and that they instead relied on peripheral cues. Secondly, when interpreted alongside the main effects for 'expectedness' and the low overall frequency values (see Supplementary Figure 1), these data indicate that saccades were mostly serving to aid continuous foveal tracking of the ball. More specifically, it appears that the 'predictive' group would generally pursue the ball with high levels of coherence, but might occasionally be required to employ small catch-up saccades to facilitate these processes (e.g., when balls were projected from an unexpected location).

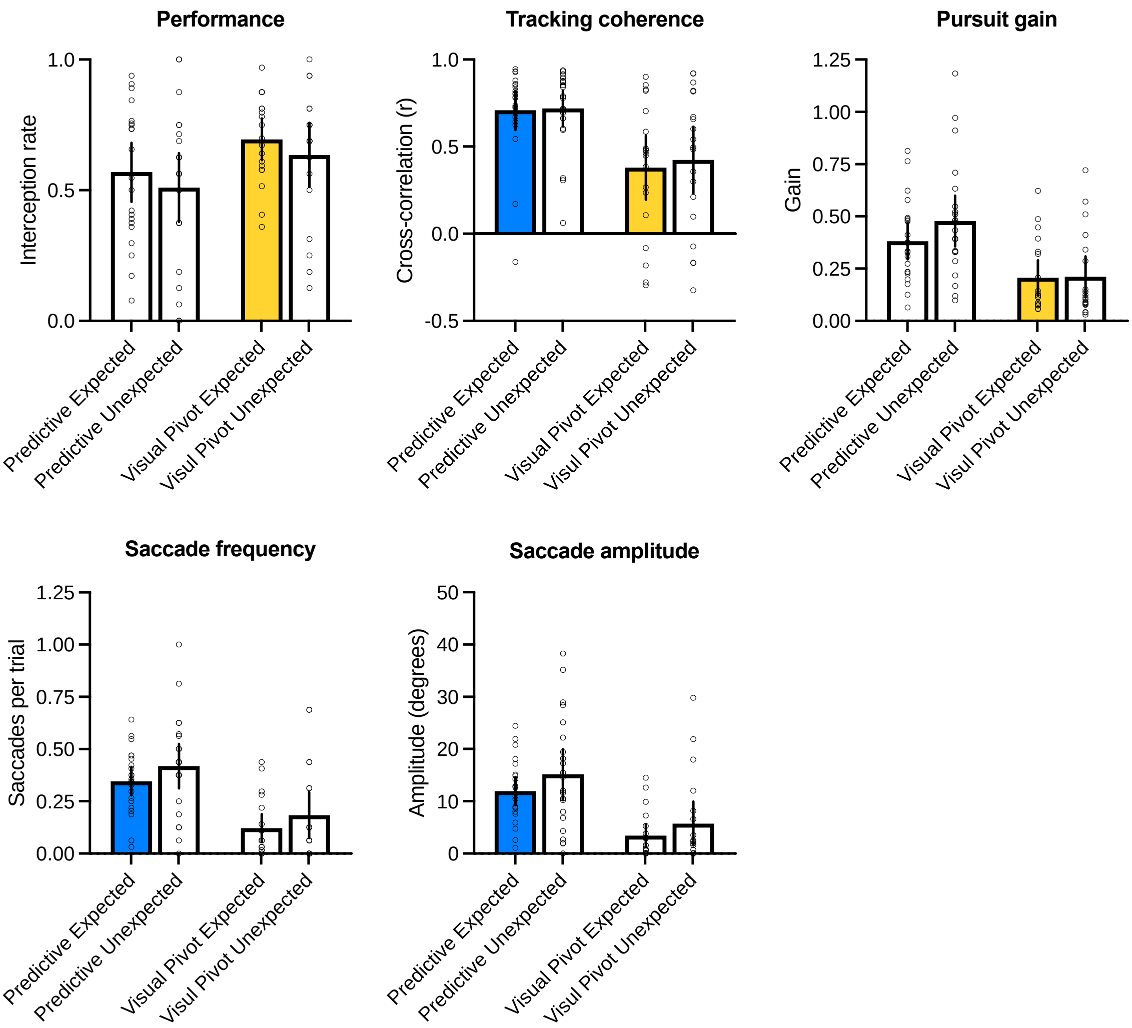

Supplementary Figure 1 – comparisons of performance and tracking behaviours of the two strategy groups for expected and unexpected trials.
